# Investigation of the global transportation of *Culicoides* biting midges, vectors of livestock and equid arboviruses, from flower‐packing plants in Kenya

**DOI:** 10.1111/mve.70016

**Published:** 2025-10-08

**Authors:** Jessica Eleanor Stokes, Karien Labuschagne, Eric Maurice Fèvre, Matthew Baylis

**Affiliations:** ^1^ Liverpool University Climate and Infectious Diseases of Animals (LUCINDA) Group, Institute of Infection, Veterinary and Ecological Sciences University of Liverpool, Leahurst Campus Neston Cheshire UK; ^2^ ARC – Onderstepoort Veterinary Research, EPV Onderstepoort South Africa; ^3^ International Livestock Research Institute Nairobi Kenya; ^4^ National Institute for Health Research, Health Protection Research Unit in Emerging and Zoonotic Infections University of Liverpool Liverpool UK

**Keywords:** African horse sickness virus, arbovirus, bluetongue virus, *Culicoides*, flowers, Kenya, Schmallenberg virus, transport

## Abstract

In recent decades there has been a huge increase in the export of cut flowers from countries in Africa and elsewhere to European flower markets, with the vast majority first entering the Netherlands for local use or for export. Coincidentally, three significant livestock disease outbreaks caused by viruses associated with Africa or other tropical regions were first detected in the Netherlands (bluetongue virus serotype 8 (BTV‐8), 2006, and BTV‐3, 2023) and in western Germany about 200 km from the Netherlands border (Schmallenberg virus, SBV, 2011). This study aimed to determine whether *Culicoides* biting midges (Diptera: Ceratopogonidae), the vectors of BTV and SBV, are present within flower‐packaging plants in East Africa, and therefore whether *Culicoides* could be unknowingly exported during the shipping of cut flowers. Field sampling was undertaken at a flower‐packaging facility in Kenya, East Africa. The facility undertook all stages of cut flower production from maintaining rootstock through to packaging and shipping to an airport for international export. Trapping was undertaken at each stage of production (rootstock, propagation, inside growing greenhouses, in the packing‐house, inside cold‐storage rooms, during transportation) using Centers for Disease Control and Prevention (CDC) Light Emitting Diode (LED) light traps. Hand‐held aspirators were used to obtain individual insects directly from flowers and around composting sites, while emergence traps studied insect emergence from compost, leaf litter and flowers discarded at quality control checkpoints. A maximum nightly catch of 269 *Culicoides* was identified on a half‐acre smallholding, containing 15 ruminants and 40 birds, located 20 m from the nearest greenhouse. The greatest numbers of *Culicoides* were trapped at a pond (*n* = 23) and leaf‐litter compost site (*n* = 19) within the curtilage of the flower‐packaging plant. Of the seven greenhouses sampled, three had *Culicoides* trapped overnight (mean = 4, range: 1–9), and no *Culicoides* were trapped in the propagation units. No *Culicoides* were trapped in the pack house, cold‐store, or during transportation of the flowers to the airport for shipment. No *Culicoides* emerged from emergence traps or were trapped when aspirating directly from flowers. This is the first study to investigate whether *Culicoides* are present within flower packaging plants in Africa. The results highlight that although present in small numbers both outside and within greenhouses, the presence of *Culicoides* declined with each stage of production. Therefore, the risk of exporting *Culicoides* with packaged cut flowers is non‐zero but likely very small.

## Graphical abstract

- Arboviral diseases spread by *Culicoides* biting midges have been introduced into Europe by unknown means. A possible route is the carriage of midges with cut flowers shipped to flower markets.
- We sampled Culicoides in and around a cut flower farm in Kenya; midges were caught in the vicinity and a greenhouse, but not where flowers are processed.
- The chance of Culicoides being transported to Europe with cut flowers is extremely low, but non‐zero.

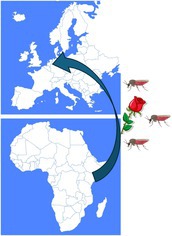

## BACKGROUND

Biting midges of the genus *Culicoides* (Diptera: Ceratopogonidae) transmit several important viruses of ruminants, such as bluetongue virus (BTV), epizootic haemorrhagic disease virus and Schmallenberg virus (SBV), as well as African horse sickness virus (AHSV) (Mellor et al., 2000). Prior to 1998, BTV was considered exotic to Europe, with subsequent incursions of several BTV serotypes into southern Europe between 1998 and 2006, limited in distribution to the Mediterranean Basin where the main Afro‐Asiatic vector, *Culicoides imicola* Kieffer 1913, was present (Mellor et al., 2008). However, in August 2006, a BTV serotype 8 (BTV‐8) outbreak was detected in Limburg Province in southern Netherlands (Elbers et al., 2008), from where it quickly spread throughout Europe, demonstrating the potential for European Palearctic *Culicoides* species to sustain and propagate outbreaks. The BTV strain occurred 900 km further north than the northernmost limit of previous European BTV incursions and entirely bypassed southern Europe. Full genome sequencing of the virus indicated a sub‐Saharan origin (Maan et al., 2008).

In 2011, a novel *Culicoides*‐borne disease was detected near the eponymous town of Schmallenberg in western Germany, only ~200 km from the original point of detection of BTV‐8 5 years earlier (Hoffmann et al., 2012). Schmallenberg disease causes mild clinical signs in adult cattle and sheep but can cause congenital malformations and stillbirths in both species if infection coincides with the vulnerable stages of gestation (Wernike et al., 2014). The causative agent SBV, was a novel Simbu serogroup virus from the genus *Orthobunyavirus*, with sequence analysis suggesting SBV is a reassortant of Shamonda and Sathuperi viruses (Yanase et al., 2012). Although some orthobunyaviruses had previously been reported in Europe, viruses from the Simbu serogroup had not previously been isolated in the region but are widespread in other parts of the world (Saeed et al., 2001).

There have recently been further incursions of BTV into the Netherlands. In September 2023, BTV‐3 was detected in sheep southeast of Amsterdam, close to Schipol, the main international airport in the Netherlands (Holwerda et al., 2024). In October 2024, BTV‐12 was detected in a sheep, close to where BTV‐3 had first been detected (van den Brom et al., 2025). The origins of these viruses are unknown at present.

The emergence of these viruses highlights the need for a greater understanding of potential incursion routes of *Culicoides*‐borne viruses into northern Europe. An investigation of possible entry routes of BTV‐8 into northern Europe in 2006 considered the legal or illegal import of infected animals; the import of biological materials potentially containing the virus (vaccines, serum, semen); or the import of infected *Culicoides*, either on the wind, in aircraft, or packed up with plants (Mintiens et al., 2008). The study failed to identify the route conclusively but suggested that the potential import of infected *Culicoides* should be further studied.

Here, we investigate the potential for *Culicoides* to be imported to northern Europe with cut flowers. Flowers exported from Africa to Europe are grown in greenhouses, packed at night under bright lights (Mintiens et al., 2008), wrapped in polythene, chilled, and transported by air. It is theoretically possible that *Culicoides* could be packed up with the flowers, survive the journey, and be released after arrival. Routine monitoring of pests in exported plants is not undertaken either by farms or by exporters as it would disrupt the just‐in‐time supply chain for fresh products such as flowers, and export controls are largely risk‐based.

The international trade in cut flowers has grown dramatically in recent years, with an export value of $10 bn in 2022 (Hinsley et al., 2025). The flowers are primarily sourced from Latin America and Africa and supply markets in North America and Europe. In Europe, the Netherlands is a hugely significant hub with, in 2014, about 60% of all imports of cut flowers to the European Union entering the Netherlands first, before either local purchase or export to other countries (Anon, 2016). For example, in 2017 the Netherlands imported nearly 273 thousand tons of cut flowers. Pests are regularly found in consignments: an investigation of cut flowers imported into Australia found about 40% of consignments from Kenya harboured pests (Anon, 2019). An analysis of 1538 interceptions into the UK and the Netherlands in 2017 identified 459 different pest types, about 90% insects and ~6% arachnids (Hinsley et al., 2025). The vast majority were plant pests, such as white fly, noctuid moths, leaf miners, thrips and aphids. However, it is noteworthy that in the Netherlands both Culicidae (the family of mosquitoes) and Nematocera (a suborder of Diptera, that includes mosquitoes and midges) were listed among identified pests.

This study aims to test the hypotheses that female *Culicoides* (1) can be found within flower‐packaging plants in Kenya supplying goods to global flower markets; (2) are found during each stage of the flower production and packaging process; and (3) are found during transportation from flower‐packaging plants to the airport.

## METHODS

### Field site

Field sampling was undertaken during October 2014 at a flower‐growing and packaging facility in Kenya, East Africa, at 1500 m altitude. A previous study sampling *Culicoides* in Kenya indicated that peak numbers are generally caught in April to May, after the long rains, but they are present year‐round and can be found at least to an altitude of 2000 m (Walker & Davies, 1971). The precise location of the farm cannot be disclosed, to protect commercial interests. The farm undertook all stages of flower production from maintaining rootstock and outdoor crops, to propagation of young stock and growing on of established plants, to packaging flowers to individual customer requirements and exportation of packaged products using cold‐storage methods. Flowers which did not meet quality control expectations were composted on site. Although more than one variety of flower was grown, the farm specialised in the exportation of roses into the European market, to both auction houses as well as directly to supermarkets and wholesalers. More than 20 greenhouses were used to grow roses on site, varying in size between a 0.3 hectare ‘show’ greenhouse, used to show customers the full variety of roses grown on site, up to 2.7 hectare greenhouses for growing on the most popular rose varieties. The total size of the greenhouses was 60 ha.

### Field sampling

Trapping was undertaken over eight nights using Centers for Disease Control and Prevention (CDC) Light Emitting Diode (LED) light traps connected to a 6‐volt battery. Sixteen sites were selected to represent each stage of production (rootstock, propagation, inside growing greenhouses, in the packaging‐house, inside cold‐storage rooms, during transportation). Each site was sampled for 2 nights (with 4 sites sampled each night over the 8‐night period). Hand‐held aspirators were used to obtain individual insects directly from flowers and around composting sites, while four emergence traps, in place for five nights, were used to capture any freshly emerging insects from compost, leaf‐litter and flowers discarded at quality control checkpoints. Details of the type of production system, as well as the use of any insecticides, and biological and mechanical control methods used within each greenhouse were also recorded.

### Smallholdings

Additionally, two smallholdings (hereafter referred to as SH1 and SH2) that directly neighboured the facility had CDC traps present for 3 nights each. SH1 was located 270 m from the nearest greenhouse at the flower farm and contained 10 dairy cattle, a small number of dairy goats, and 30 rabbits on 2.5 ha. The majority of SH1 was used for field crops supplied by drip irrigation and vermi‐culture compost. SH2 was 20 m from the nearest greenhouse and contained 15 ruminants and 40 birds on a 0.38 hectare site.

### Specimen identification

*Culicoides* were separated from other insects according to their wing characteristics using a stereomicroscope and stored in 70% ethanol. *Culicoides* were sexed based on their antennae and identified to species level based on their wing marking and other characteristics (Glick, 1990); other insects were mostly identified to the level of family or order.

## RESULTS

### Insects trapped

The 38 trap catches produced a total of 445 *Culicoides* of 13 species, and 2617 other insects of 26 genera over the eight nights of trapping. The single largest nightly catch of *Culicoides* was 269 at SH1 (140 *C. imicola* (3 male), 91 *C. bolitinos* Meiswinkel 1989, 25 *C. enderleini* Cornet and Brunhes 1994 (2 male), 10 *C. brucei* Austen 1909 and one each of *C. grahamii* Austen 1909, *C. miombo* Meiswinkel 1991 and *C. kibatiensis* Goetghebuer 1935; all female unless specified), while the second largest catch was 107 at SH2 (87 *C. imicola* (3 male), 10 *C. bolitinos*, 5 *C. nivosus* de Meillon 1937 (1 male), 3 *C. enderleini* (3 male) and one each of *C. brucei* and *C. similis* Carter, Ingram and Macfie 1937). In the facility itself, the single largest nightly catch was 23 *Culicoides* (17 *C. similis*, 2 *C. imicola*, 3 *Culicoides trifasciellus* Goetghebuer 1937 (1 male) and 1 *C. neavei* Austen 1912) which were trapped near a pond behind one of the greenhouses, followed by 19 *Culicoides* (6 *C. trifasciellus*, 5 *C. bedfordi* Ingram and Macfie 1923 (1 male), 3 *C. imicola*, 4 *C. enderleini* (1 male) and 1 *C. similis*) trapped at a leaf litter composting site between two greenhouses. Inside the greenhouses themselves, we caught 9 and 2 *Culicoides* in two of the eight greenhouses. These comprised 7 *C. similis*, 1 *C. imicola*, 1 *C. pycnostictus* Ingram and Macfie 1925 (all female) and one male each of *C. enderleini* and *C. bedfordi*. No *Culicoides* were caught in the remaining sites. The most abundant species of *Culicoides* were *C. imicola* (55.4%), followed by *C. bolitinos* (24.1%), *C. enderleini* (6.6%) and *C. similis* (6.1%) (Table 1).

**TABLE 1:** *Culicoides* species trapped at a flower growing and packaging plant, and two adjacent smallholdings in Kenya.

Species trapped	Female (% of total catch)	Male (% of total catch)
*Culicoides bedfordi*	5 (1.2)	2 (9.5)
*Culicoides bolitinos*	102 (24.1)	0
*Culicoides brucei*	11 (2.6)	0
*Culicoides enderleini*	28 (6.6)	9 (42.9)
*Culicoides grahamii*	1 (0.2)	0
*Culicoides imicola*	235 (55.4)	7 (33.3)
*Culicoides kibatiensis*	1 (0.2)	0
*Culicoides miombo*	1 (0.2)	0
*Culicoides neavei*	1 (0.2)	0
*Culicoides nivosus*	4 (0.9)	1 (4.8)
*Culicoides pycnostictus*	1 (0.2)	1 (4.8)
*Culicoides similis*	26 (6.1)	0
*Culicoides trifasciellus*	8 (1.9)	1 (4.8)
Total	**424 (100)**	**21 (100)**

Blood‐fed individuals of *C. imicola* and *C. bolitinos* were trapped solely on the smallholdings. All *C. imicola* trapped on the flower farm were gravid. No *C. bolitinos* were trapped on the flower farm.

Of the other insects trapped, the single largest catch (*n* = 857) was also at SH2, with the second largest catch from near a pond behind the greenhouses (*n* = 530). Non‐*Culicoides* were trapped at every sampling location (including inside every greenhouse). The largest single catch inside a greenhouse was 97 non‐*Culicoides*. This was the same trapping sample that produced the single largest catch of *Culicoides*. No *Culicoides* were trapped from aspirating directly from plants or from the emergence traps.

Members of the Family Psychodidae were the most abundant non‐*Culicoides* trapped (*n* = 683; 26.10% of other insects), followed by the Family Chironomidae (*n* = 515; 19.68%) (Table 2).

**TABLE 2:** Other arthropods trapped at a flower growing and packaging plant, and two adjacent smallholdings, in Kenya.

Class	Order	Family	Common name	Number	% of total
Insecta	Coleoptera		Beetle	395	15.1
	Collembola		Springtail	29	1.1
	Diptera	Drosophilidae	Vinegar fly	3	0.1
		Chironomidae	Non‐biting midge	515	19.7
		Culicidae	Mosquito	14	0.5
		Other Ceratopogonidae		25	1
		Other Diptera		32	1.2
		Other Nematocera		369	14.1
		Phoridae	Coffin fly	30	1.2
		Psychodidae	Sewer flies	683	26.1
		Tipulidae	Crane fly	54	2.1
			Filth fly	5	0.2
			Fungus gnat	145	5.5
	Hemiptera		Aphid	9	0.3
	Other Hemiptera		87	3.3	
	Hymenoptera		Ant	19	0.7
			Sawfly	1	0.04
			Wasp	19	0.7
	Lepidoptera		Moth	32	1.2
	Neuroptera		Lacewing	3	0.1
	Psocoptera		Booklice	47	1.8
	Thysanoptera		Thrips	44	1.7
Arachnida	Acari		Mite	43	1.6
	Aranea		Spider	13	0.5
			Total	**2616**	**100**

### Stages of production

Traps set outdoors captured the greatest numbers of *Culicoides* (SH1, *n* = 269; SH2 *n* = 122; near water sources, *n* = 23; composting sites, *n* = 19; rootstock, *n* = 1). Traps set in greenhouses collected a total of only 11 *Culicoides*, while traps set in the packaging room, cold storage, and transportation captured no *Culicoides* at all. Similar results were seen for the non‐*Culicoides* trapped, with the largest numbers captured in outdoor environments, and fewer captured as the stages of production neared cold storage and transportation (Table 3).

**TABLE 3:** *Culicoides* and other insects trapped at each stage of production on a flower growing and packaging plant in Kenya.

Production stage	Number of *Culicoides* (% of total catch)	Number of non‐*Culicoides* (% of total catch)
Smallholding 1	269 (60.5)	918 (35.1)
Smallholding 2	122 (27.4)	454 (17.4)
Near water sources	23 (5.2)	530 (20.3)
Composting	19 (4.3)	185 (7.1)
Rootstock/outdoor crops	1 (0.2)	90 (3.4)
Indoor propagation	0	91 (3.5)
Greenhouses	11 (2.5)	177 (6.8)
Packaging room	0	166 (6.3)
Cold storage room	0	0
Transportation	0	6 (0.2)
Total	**445 (100)**	**2617 (100)**

### Disease and pest control

**FIGURE 1:**
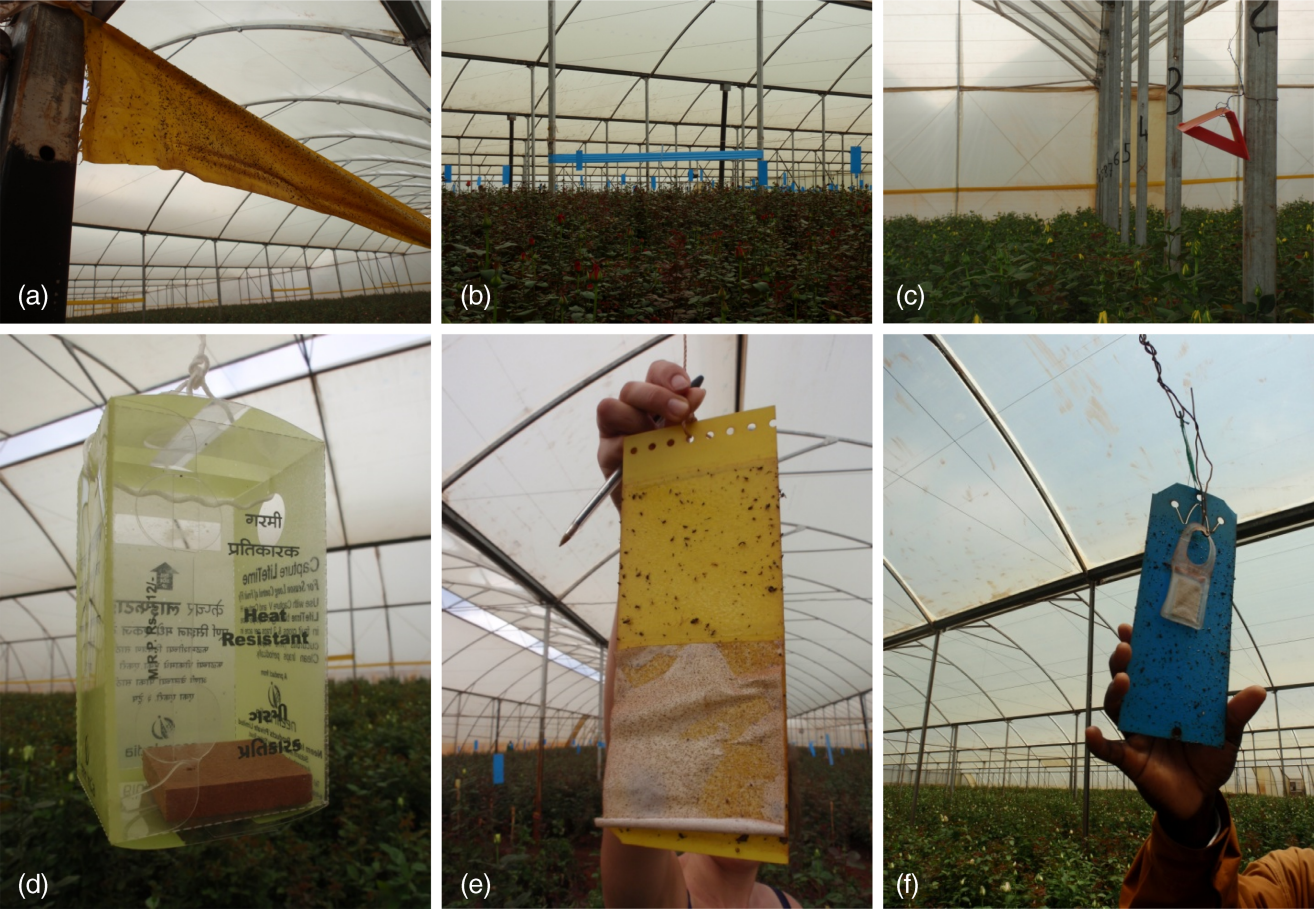
Methods of mechanical pest control undertaken within the greenhouses at a flower‐packaging plant in Kenya. (a) Roller trap for white flies, aphids, leaf miners; (b) roller trap for thrips; (c) moth and butterfly traps; (d) fruit fly traps; (e) pest‐level monitoring traps; and (f) scented thrips traps.

Both mechanical and chemical pest control methods were undertaken at the site. Three of the greenhouses sampled had mechanical pest control options in place. These included roller traps for white flies, aphids, leaf miners, and thrips; moth and butterfly traps, fruit fly traps, pest‐level monitoring traps, and scented thrips traps (Figure 1). Several of the roller traps were examined in situ using hand lenses by GK and JES, and no *Culicoides* were observed. Air circulation systems were in place, and insecticides were sprayed overnight in greenhouses depending on what pests were found to be present. A number of the greenhouses were also used to test biological control methods for pests. Of the greenhouses sampled, three of them were sprayed with insecticide the night before trapping took place, with three greenhouses recording details of known pests and diseases present (Table 4).

**TABLE 4:** The eight growing greenhouses and one propagation greenhouse sampled at a flower farm in Kenya.

Greenhouse	Ha	Hydroponic	Mechanical pest control	Insecticide usage	Rose varieties	Pests	Diseases
Propagation	0.4	No	No	No	Multiple	‐	‐
GH4	2	No	Yes	No	2	RSM, MB, TH	PM, DM, PB
GH5	2	Yes	Yes	Yes	4	‐	‐
GH7	2.3	No	No	No	3	‐	‐
GH10	0.8	No	Yes	Yes	1	C, MB, TH, M	PM, DM, PB, AB
GH11	0.3	Yes	No	No	Multiple	‐	‐
GH17	1.4	Yes	No	No	2	AP, TH, C, RSM, MB	PM, DM, PB, AB
GH19	1.45	No	No	Yes	2	‐	‐
GH23	0.72	No	No	No	Newly planted	‐	‐

*Note*: Details given on: The number of hectares occupied by the greenhouse (Ha); whether hydroponics or mechanical pest management methods were employed; whether the greenhouse was sprayed with insecticide prior to sampling; the pests (AP, aphids; C, caterpillars; M, mites; MB, mealybugs; RSM, red spider mite; TH, thrips) and diseases (AB, agrobacteria; DM, downy mildew; PB, petal botrytis; PM, powdery mildew) to be controlled.

## DISCUSSION

This study is the first to investigate whether *Culicoides* biting midges are present on flower farms and packaging plants in Africa. The results demonstrate that *Culicoides* could be found in substantial numbers on smallholdings adjacent to the flower farm, and that small numbers of *Culicoides* were caught in the greenhouses themselves. However, none were trapped where cut flowers were packaged for export. The species caught in the smallholdings and greenhouses included the major Afrotropical vector of several arboviruses, *C. imicola*; while an established vector of BTV and AHSV, *C. bolitinos*, was trapped in the smallholdings but not in the greenhouses. We failed to trap any *Culicoides* at all in the areas where cut flowers were prepared for export. However, given the scale of the flower trade from Africa to Europe (Anon, 2016), our results are consistent with there being a very small, but non‐zero risk of biting midges occasionally being transported along with the produce.

### Route of introduction to Europe

There are several hypotheses as to the possible introduction of *Culicoides* into northern Europe in 2006 (Mintiens et al., 2008). One theory is that infected *Culicoides* were transported into the Maastricht area of the Netherlands with cut flowers from Africa. The insects are hypothesised to have either entered consignments during packaging or shipping, or to have been phytotelmatic species which breed exclusively in container‐habitats, including water in the leaf axils of plants. Only 5% of *Culicoides* species are phytotelmatic; however, of those species, only *C. loughnani* and possibly *C. paolae*/*C. jamaicensis* are believed to have a substantiated record of long‐distance introduction on plants (Dyce, 1969; Meiswinkel, Labuschagne, & Goffredo, 2004). This contrasts with other insects such as the mosquito *Aedes albopictus*, where their worldwide transportation, in lucky bamboo plants (*Dracaena* spp.) imported from south‐east Asia, is well documented (Ibáñez‐Justicia et al., 2020). Even if there is phytotelmatic transport of juvenile *Culicoides*, the appearance of BTV‐8 in northern Europe remains enigmatic as, in the absence of documented vertical transmission of BTV, only adult female *Culicoides* can be infected with such viruses.

Kenya is the third largest flower exporter globally, after Colombia and Ecuador, and is the leading exporter of cut roses to the EU (38%) (Anon, 2016). Of these flower consignments, almost 65% are sold through Dutch auction houses (Anon, 2019), with the industry seeing a growth of 5% each year. The EU absorbs 85% of all flower exports from Kenya (Anon, 2014), with its main cut flower export being roses (Anon, 2016), followed by *Dianthus caryophyllus* (carnations) and *Alstroemeria* (Peruvian lily). A Kenyan rose producer and exporter is, therefore, the ideal field site for assessing whether female *Culicoides* can be found within flower packaging plants, as well as the level of risk of *Culicoides* being transported in cut flowers to Europe.

### Environment at a flower farm

An important aspect of the current study is that midges were trapped at different stages of the production process, which highlights that although *Culicoides* are present around the flower farm, their numbers diminish with each stage of the production process, therefore reducing the likelihood of transporting them with each stage of the process.

According to (Mintiens et al., 2008), consignments of plants are usually packed in the country of origin at night, when conditions (lighting, humidity) during packaging would appear favourable for incorporating insects during packaging, and plants are watered prior to shipment. This was not the case for the flower farm in Kenya where this trapping took place. During daytime, cut flowers were held in water until packaged, and were then stored in a sealed room at 4°C until transportation to the airport. *Culicoides* are inactive at such low temperatures (Mellor et al., 2000) and this would make the incorporation of midges in the packages unlikely, unless they were already present on the flower when taken to the room. The flowers were transported in refrigerated lorries directly to the airport prior to shipment. No flowers were processed or transported at night. We do not know if this is now standard practice in Kenya, but in our view, this approach presents a lower risk of transporting *Culicoides* than preparing cut flowers at night.

### Culicoides trapped

Of the over‐50 arboviruses associated with *Culicoides*, 15 have been isolated from species belonging to the *C. imicola* complex (Mellor et al., 2000). The *C. imicola* complex (*C. imicola*, *C. bolitinos*, *C. brevitarsis*, *C. nudipalpis*, *C. kwagga*, *C. loxodontis*, *C. miombo*, *C. pseudopallidipennis*, *C. tutti‐frutti* and *C. asiana*) contains the three most important known vectors of bluetongue virus: *C. imicola*, *C. bolitinos* and *C. brevitarsis* (Meiswinkel, Gomulski, et al., 2004). Both *C. imicola* and *C. bolitinos* were trapped during this study. *Culicoides bolitinos* was trapped exclusively on both smallholdings outside the flower farm, likely due to its strong association with cattle dung as a breeding site. *Culicoides imicola* was trapped at both smallholdings, but was also trapped near to a pond, at a composting site, and inside two greenhouses on the flower farm. This may be an indication of their need for semi‐moist soil breeding sites, as opposed to the dung sites *C. bolitinos* occupy.

## CONCLUSIONS

While we did not detect any *Culicoides* within the facility where cut flowers are prepared for export, we did detect low numbers of several species in the greenhouses where the flowers are grown, including some individuals of an important arbovirus vector. Significant numbers of *Culicoides*, including two vector species, were detected in close vicinity to the farm where livestock were kept. While not directly evidenced, our data are consistent with a small but non‐zero risk of *Culicoides* vectors occasionally being transported along with cut flowers. Given the scale and value of the international industry in flower exports, and the potential risks to importing countries, we recommend simple interventions that reduce risks and protect the industry. This should include light traps for biting midges in the processing rooms of flower farms and importing airports. We further recommend that flower farms work with their smallholder neighbours to remove *Culicoides* breeding sites and/or hang midge‐proof netting (with a minimum 1000 holes per square inch) on the side of greenhouses close to smallholdings.

## AUTHOR CONTRIBUTIONS

**Jessica Eleanor Stokes:** Investigation; writing – original draft; writing – review and editing; formal analysis; methodology. **Karien Labuschagne:** Writing – original draft; writing – review and editing; formal analysis; data curation. **Eric Maurice Fèvre:** Methodology; conceptualization; funding acquisition; writing – original draft; writing – review and editing. **Matthew Baylis:** Conceptualization; methodology; supervision; funding acquisition; writing – original draft; project administration; writing – review and editing.

## CONFLICT OF INTEREST STATEMENT

The authors declare no conflicts of interest.

## Supporting information


**Data S1.** Structured reflexivity statement.

## Data Availability

Data used in this manuscript are available from the University of Liverpool repository, https://doi.org/10.17638/datacat.liverpool.ac.uk/3034 (Baylis et al., 2025).
