# Supplementary material for: Investigation of the global transportation of Culicoides biting midges, vectors of livestock and equid arboviruses, from flower‐packing plants in Kenya
Source: Med Vet Entomol. 2025 Oct 8;40(2):260–7. doi: 10.1111/mve.70016 (PMC13140018; doi:10.1111/mve.70016)
Supplement: Supplementary file 1 — Data S1. Structured reflexivity statement. [file MVE-40-260-s001.docx]

Please complete all sections relevant to your research study

| Study conceptualisation | |  |
| --- | --- | --- |
| Please justify the choice to conduct the study in and/or acquire samples from the LMIC location | | The study was investigating possible movement of Culicoides from flower farms in East Africa – hence the study had to be done there (Kenya). |
|  | How does this study address local research priorities and how were local researchers involved in study design? | The flower trade is important for the Kenya economy and risks to it and from it are of importance. The study involved 2 authors based in Europe and 2 in Middle Income Countries (Kenya and South Africa). One author (Kenya) helped to write the funding proposal and provided the link to the flower farm. |
| Research management | |  |
|  | How has funding been used to support the local research team(s)? | No funding was made available to the local research team or South African researcher. Funding was used exclusively to support 2 UK students to visit Kenya and stay on a flower farm to collect the samples. |
| Data acquisition and analysis | |  |
|  | How are research staff who conducted data collection acknowledged? | Stokes was a PhD student at the time and is the lead author. The other person who directly collected the data declined authorship but is acknowledged. |
|  | How have members of the research partnership been provided with access to study data and analytical tools? | All members have access to the data. |
|  | If genetic resources were shared, how were the principles of the Nagoya Protocol on the equitable sharing of benefits | No genetic resources were taken out of Kenya. All genetic material was disposed of at the end of the study. |
| Data interpretation | |  |
|  | How have research partners collaborated in interpreting study data? | All have been involved. |
| Drafting and revising for intellectual content | |  |
|  | How were research partners supported to develop writing skills? | All have contributed to the final manuscript |
| Authorship | |  |
|  | How is the leadership, contribution and ownership of this work by LMIC researchers recognised within the authorship? | All included as authors |
|  | How have early career researchers across the partnership been included within the authorship team? | Stokes was a PhD student and is lead author. |
|  | How has gender balance been addressed within the authorship? | The authorship is 2 x female and 2 x male. |
| Training | |  |
|  | How has the project contributed to training of LMIC researchers? | LMIC-based researchers were already experienced. However, the South African Culicoides expert gained direct experience of identifying midges from Kenya. |

Adapted from: Morton et al. 2021 “Consensus statement on measures to promote equitable authorship in the publication of research from international partnerships” <https://doi.org/10.1111/anae.15597>
